# Do Parents Meet Adolescents’ Monitoring Standards? Examination of the Impact on Teen Risk Disclosure and Behaviors if They Don’t

**DOI:** 10.1371/journal.pone.0125750

**Published:** 2015-05-08

**Authors:** Lesley Cottrell, Carrie Rishel, Christa Lilly, Scott Cottrell, Aaron Metzger, Halima Ahmadi, Bo Wang, Xiaoming Li, Bonita Stanton

**Affiliations:** 1 Department of Pediatrics, West Virginia University School of Medicine, Morgantown, WV, United States of America; 2 School of Social Work, West Virginia University, Morgantown, WV, United States of America; 3 Department of Biostatistics, West Virginia University School of Public Health, Morgantown, WV, United States of America; 4 Department of Medical Education, West Virginia University School of Medicine, Morgantown, WV, United States of America; 5 Department of Psychology, West Virginia University, Morgantown, WV, United States of America; 6 Department of Social and Behavioral Sciences, West Virginia University School of Public Health, Morgantown, WV, United States of America; 7 Pediatric Prevention Research Center, Department of Pediatrics, Wayne State University, Detroit, MI, United States of America; 8 Wayne State University, School of Medicine, Detroit MI, United States of America; Swansea University, UNITED KINGDOM

## Abstract

In this study, we examined how adolescents compare monitoring efforts by their parents to those of a "good parent" standard and assessed the impact of these comparisons on adolescent self-disclosure and risk behavior and their perceptions of their parents' monitoring knowledge. Survey responses from 519 adolescents (12–17 years) at baseline of a larger, longitudinal study examining parental monitoring and adolescent risk were examined. Adolescents’ “good parent comparisons” differed greatly by monitoring areas (e.g., telephone use, health, money); however, between 5.5% and 25.8% of adolescents believed their parents needed to monitor their activities more than they currently were monitoring. Alternatively, between 8.5% and 23.8% of adolescents believed their parents needed to monitor their activities less often. These perceptions significantly distinguished adolescents in terms of their level of disclosure, perceived monitoring knowledge, and risk involvement. Adolescents who viewed their parents as needing to monitor more were less likely to disclose information to their parents (p<.001), less likely to perceive their parents as having greater monitoring knowledge (p<.001), and more likely to be involved in a risk behaviors (p<.001) than adolescents who perceived their parents needed no change. Adolescent disclosure to a parent is a powerful predictor of adolescent risk and poor health outcomes. These findings demonstrate that adolescents' comparisons of their parents' monitoring efforts can predict differences in adolescent disclosure and future risk. Obtaining adolescent "good parent" comparisons may successfully identify intervention opportunities with the adolescent and parent by noting the areas of need and direction of monitoring improvement.

## Introduction

Over the past several decades, parents or legal guardians of adolescents have been extensively examined with regard to their contributions to adolescent delinquency. Prior to the new millennium, parents’ composite knowledge about their adolescents’ friends, activities, and whereabouts (i.e., parental monitoring knowledge) was the fundamental factor studied for its inverse association with adolescent risk involvement [1–3]. In 2000, Kerr and Stattin re-examined the parental monitoring construct based not only on the *amount* of information parents obtain about their adolescents but also *how* they obtained that information [4]. From this expanded perspective, the most powerful predictor of parental monitoring knowledge was adolescent self-disclosure of information [4–5]. This research produced a surge of studies questioning the direct role parents had on adolescent delinquency and the way in which parental monitoring was measured [6–8].

The realignment of the parental monitoring literature resulted in a series of subsequent studies primarily focused on adolescent self-disclosure. This research approach continues to examine parenting factors based on their impact on adolescent self-disclosure. Monitoring strategies that are direct, express responsiveness and have low psychological control are associated with greater adolescent self-disclosure than monitoring strategies involving high psychological control and limited closeness [9–12]. Despite what we know about how parents monitor, who perceives and reports those monitor efforts also matters. Parent and adolescent perceptions of parents’ monitoring efforts are not often similar in nature and frequency [13–14]. When different, adolescent perceptions of monitoring are often more strongly associated with their engagement in risk behaviors than parent perceptions [14].

Differences in parent and adolescent monitoring views could be accounted for by the different sets of expectations and contrast cognitive framing that Collins [15] and Smetana [16] demonstrated were present in parent-adolescent views of conflict. Likewise, elements can be drawn from the Social Comparison Theory [17], particularly those noting the use of information from others to assess one’s own behaviors, to begin to explore potential processes contributing to different parent and adolescent expectations and reports of monitoring. Currently, little information is known about adolescent expectations of their parents’ monitoring efforts and whether these expectations may be associated with the extent to which adolescents self-disclose to their parents, perceive their parents to have greater overall parental monitoring knowledge, and/or engage in risk behaviors. Given that adolescent perceptions of their parents’ monitoring knowledge is more strongly associated with their risk behaviors, it would be important to explore adolescents’ perceptions of their parents’ monitoring compared to a perceived standard. Adolescents may compare their parents’ monitoring efforts to a perceived social standard, and, if divergent from such standard, they may be less likely to self-disclose, more likely to perceive their parents’ monitoring to be limiting, and more likely to engage in risk behaviors. Adolescents’ social comparisons may be different from those of their parents’ creating a divergence in perceptual lenses within the family about parents’ monitoring and its impact. Before we answer these and other questions, we must first explore this idea of adolescent social comparisons with regards to their parents’ monitoring.

In the present study, we investigated the extent to which adolescents view their parents’ monitoring efforts compared to those of a “good parent” among a cross-sectional sample of adolescents (12–17 years). We also explored whether adolescent comparisons differed based on select individual factors including adolescent age, gender, and family income. To complete this initial assessment of adolescents’ comparisons, we tested the impact of these adolescent comparisons to a “good parent” standard on factors associated with adolescent risk behavior including adolescent report of self-disclosure, amount of parental monitoring knowledge, and actual report of risk behaviors in the past four months.

## Materials and Methods

### Study Population

Five hundred nineteen adolescent-parent dyads (39% of eligible students) participated in this study. Adolescents were between 12 to 17 years of age (mean age = 15 years). Over two-thirds of the adolescents (68.5%) were female and lived with a biological parent (91.1%). The majority of the sample was Caucasian, non-Hispanic (93.8%) and represented the racial and ethnic composition of the state in which they lived. Slightly less than half (47.4%) of the sample had two or more siblings/ stepsiblings living with them. By study design, one parent participated with each adolescent in this study. Mean parent age was 37 years. Eighteen percent of adolescents were reportedly from households with incomes less than $15,000. Adolescents were recruited to participate in a monitoring intervention project entitled, *Communication Between Parents and Adolescents (COPA)*, in a rural setting. Their responses to the baseline survey are used in this study.

### Study Design and Measures

Adolescent comparisons of their parents’ monitoring were assessed using a “good parent comparison scale” designed specifically for this study. This scale is composed of nine items designed to assess adolescents’ views of their parents’ monitoring effectiveness in specific areas using phrases similar to, “Compared to what a ‘good parent’ might do, do you think your parent should monitor you more, less, or about the same amount as he/she does now?” Sample monitoring areas of adolescent behaviors included: telephone usage, general appearance, mood, schoolwork and performance, and activities and plans with friends.

Adolescent response options included “more” (1), “less” (2), or “about the same” (3). These items were examined separately and as a summed score in this study. Cronbach’s alpha for the composite scale was 0.88. The scale ranged from 9 to 27. For some analyses, the total sums equal ≤ 16 were categorized as representing the group of adolescents who believed their parents needed to monitor them more. Scale scores between 17 and 26 represented the group who perceived less monitoring was needed and scores equal to 27 represented views that parental monitoring was about the same. These categories were defined based on the distribution of adolescents’ reports. For instance, scores of 9 to 16 characterized adolescents who thought their parents should monitor “more” across all of the nine monitoring areas assessed (score of 9). Alternatively, this category might have also characterized adolescents who noted the need for “more” monitoring on the majority (at least five areas) of areas but might have chosen “less monitoring” or “about the same” for a few items thus, increasing the overall score for this variable.

Adolescent disclosure was examined using an averaged 3-item composite variable. Adolescents responded to the following items, “I tell my parent what I am doing before he/she has to ask,” “I tell my parent who I am going to be with,” and “I talk to my parent about plans with friends” based on a 4-point Likert Scale where 1 represented “strongly disagree” and 4 represented “strong agree”. Thus, the eligible range for this measure was 3 to 12. The Cronbach’s alpha reliability for this disclosure composite score was 0.75. The overall sample mean for the adolescent disclosure scale was 5.50 (SD = 1.8).

Adolescent-perceived monitoring knowledge was measured using an averaged 12-item composite scale assessing how often parents knew with whom their adolescents spent time, where they were, and what they were doing at different times throughout the day (e.g., afternoons, evenings, weekends). For each item, adolescents could choose “never” (1), “a few times” (2), “several times” (3), or “all the time” (4). The overall range for responses on the 12-item scale was 12 to 48. The Cronbach’s alpha reliability for this composite scale was 0.95. The overall sample mean for the perceived monitoring knowledge scale was 41.80 (SD = 8.1).

Adolescents were asked how frequently they engaged in a range of potentially risky or risky behaviors in the past four months. Response options for all but the sexual risk items ranged from “0 times” (1) to “5 or more times” (4). Adolescent involvement across 10 areas were included: alcohol, tobacco, marijuana and other drugs, skipped school, any suspensions from school, vandalizing behaviors, and other behaviors included staying out past curfew and working with friends to get around their parents’ rules were assessed in this study. Adolescent sexual intercourse with and without condoms as well as adolescent engagement in other sexual behaviors while still a virgin were also assessed but as dichotomized variables (yes/no) rather than in terms of frequency. A total of 13 items were combined to develop a composite risk involvement variable that was continuous by nature. In order to calculate this variable, all of the Likert scale responses were recoded into 0 times = “no” and ≥ 1 = “yes”. Once recoded, all items were summed for the composite score potentially ranging from 0 to 13. The overall sample mean for the risk composite scale was 4.66 (SD = 2.6).

### Procedures

After providing a detailed description of the purpose and design of this study, we requested permissions from middle and high school administrators representing schools throughout West Virginia to invite families to participate in the study. Adolescents of eligible ages enrolled in participating schools were give a packet including a cover letter, appropriate consent and assent forms, and a FAQ sheet about the study. Adolescents were encouraged to discuss the study with their parents and to bring back completed forms if they and one of their parents were willing to participate. Adolescents were asked to identify a parent/legal guardian who is their caregiver for most of the time (if parents were divorced, separated, or single parent). If an adolescent had two legal guardians who shared caregiving responsibilities in the same household equally, the adolescent was asked to choose only one parent who would be willing to participate in the study. Once identified, this parent was consistently referred to as the “reference parent”. Thus, adolescents were asked to answer all questionnaire items in the study with this parent/legal guardian in mind specifically.

All completed forms were confirmed with parents to ensure the signature was valid and that the parent was aware of the study. Upon consent, contact information was obtained including a mailing address. All participating adolescents were later mailed the study survey, investigator information, a return postage-paid envelope, and a reimbursement form so they could receive $25 for their completion of the survey once received. Adolescents were encouraged to complete the survey independently and to seal the envelope. Any questionnaires received without the seal or a broken seal were not included in the study (n = 2). This study is based on adolescent baseline responses in a longitudinal parental monitoring study that was conducted from 2009–2011. The procedures of the larger study are provided elsewhere [18]. This study was approved by the West Virginia University Institutional Review Board. All participants and next of kin, caretakers, or guardians provided written informed consent and assent.

### Statistical analysis

Descriptive statistics were completed for all study variables including the sample demographics to characterize the sample and investigate how adolescents compared their parents’ monitoring to the “good parent” standard. We then explored potential differences in adolescents’ “good parent comparisons” based on age, gender, and family income using a 2 x 2 x 4 factorial analysis of variance (ANOVA) with the good parent scale score as the continuous dependent variable and age (ages 12–14 vs. 15–17 years), gender (male vs. female), and family income ((< $15, $15–29,999, $30–59,000, > $60) as the independent variables. Pairwise comparisons were completed to assess differences between these groups. Finally, to examine the potential impact of adolescents’ “good parent comparisons” on their own risk disclosure to their parents, perceived parental monitoring knowledge, and risk behaviors, we conducted a MANOVA that included three levels within the “good parent comparison” variable (more, less, or about the same as “good parent”) as the independent variables and average level of adolescent disclosure, perceptions of parental monitoring knowledge overall, and composite risk involvement as the multiple dependent variables in the model. Adolescent age and family income were included as covariates in these models. Model significance was held at p≤.05.

## Results and Discussion

### Adolescents’ comparisons of parents’ monitoring efforts to a “good parent”

When asked to compare their parents’ current monitoring efforts in select areas to that of a “good parent”, most adolescents reported their parents would not need to change the amount they monitored (61.5% to 72.5% depending on area; Table 1). However, in areas related to school work and general health, close to one quarter of the adolescents in this study thought their parents needed to monitor more (24.2% and 25.8%, respectively).

**Table 1:** Parent monitoring compared to “good parent” in select areas based on adolescent report.

**Area**	**More Monitoring Needed (%)**	**Less Monitoring Needed (%)**	**About the Same Monitoring (%)**
**Telephone Use**	5.5	23.8	69.1
**Appearance**	10.6	19.2	68.5
**Mood**	15.3	19.8	63.0
**School Work/Performance**	24.2	12.3	61.5
**Activities/Plans With Friends**	12.6	22.8	62.8
**Health**	25.8	8.5	64.0
**Material Use**	6.0	19.6	72.5
**TV Use**	7.4	19.1	71.7
**Money Matters**	16.8	15.3	66.2

Other adolescents reported that their parents needed to monitor less in areas related to telephone use (23.8%), activities with friends (22.8%), and mood (19.8%). Similarly, when using the “good parent” scale score across all of these areas, our findings illustrate that a total of 50 adolescents (9.6%) believed their parents needed to do more monitoring across these areas as a whole; 84 (15.8%) adolescents believed their parents needed to monitor them less, and the remaining 387 (73.0%) perceived their parents’ monitoring to adequately compare to what a “good parent” should do.

### Adolescents’ views of their parents’ monitoring efforts based on select variables

While a majority of adolescents perceived their parents’ monitoring to be that of a “good parent”, understanding factors that may contribute to the divergent views is needed. Using a factorial ANOVA, we investigated the potential impact of adolescent age, gender, and family income on adolescents’ “good parent” views of their parents’ monitoring (scale score).

Our findings revealed main effects for adolescent age (F = 7.299, df = 1, p< .01) and family income (F = 3.36, df = 3, p< .01) on adolescents’ views of their parents’ monitoring. Specific group differences noted from Tukey pairwise comparisons revealed that younger adolescents (12–14 years), and those from more affluent families (≥ $60,000), reported more favorable views of parents’ monitoring when compared to a “good parent”. Adolescent views of their parents’ monitoring compared to a “good parent” were less favorable (i.e., should do more or less monitoring) among older adolescents (15–17 years) and those from households with lower incomes. No gender differences were noted.

### Impact of adolescents’ views of parents’ monitoring on level of disclosure, perceived knowledge and adolescent risk

**Fig 1:**
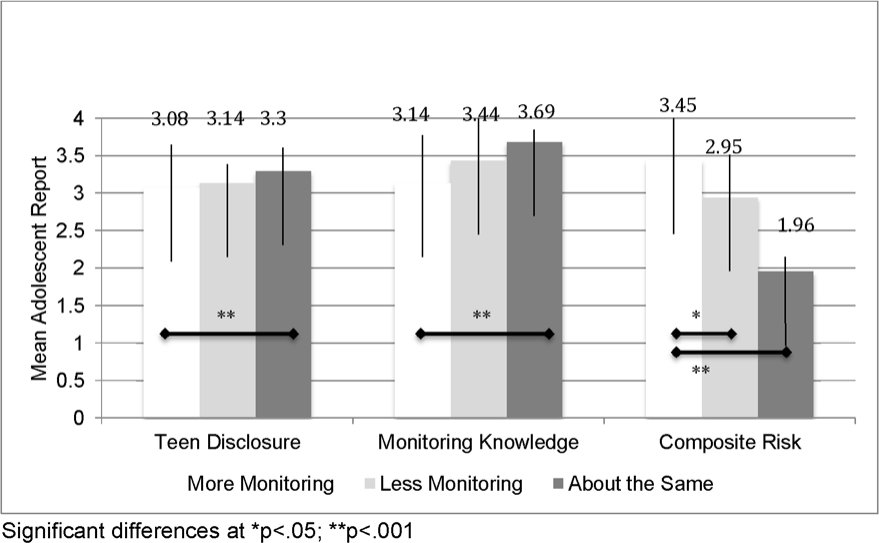
Mean adolescent disclosure and perceived parent monitoring knowledge based on monitoring comparisons to a “good parent”.

Two adolescent groups viewed their parent as needing to modify his/her parental monitoring to meet that of a “good parent”. We wanted to examine the impact of these divergent views on select outcomes including the level of adolescent disclosure and perceived extent of parent monitoring knowledge. This model was significant for adolescent disclosure (F = 7.63, df = 2, p = .001) and perceived parent monitoring knowledge (F = 19.30, df = 2, p < .001) and explained 14% and 16% of the variability in adolescent disclosure and perceived parental monitoring knowledge, respectively. Fig 1 illustrates the mean differences in adolescent disclosure and perceived monitoring knowledge based on whether adolescents perceived their parents as needing to monitor more or less than a “good parent” or maintain the same amount of monitoring. Adolescents who perceived their parents to be monitoring in a manner consistent with that of a “good parent” reported higher mean values for disclosure and parental monitoring knowledge than the remaining two groups. For both outcomes, adolescents who perceived their parents as needing to monitor more reported the lowest mean values for both disclosure and parental monitoring knowledge. Significant pairwise differences were noted between the “same as a good parent” and “more monitoring needed” for both disclosure (X = 3.30 vs 3.08, respectively; p< .001) and parental monitoring knowledge (X = 3.69 vs. 3.14, respectively; p< .000).

Significant differences in adolescent risk involvement were also found based on adolescents’ perceptions of their parents’ monitoring efforts to the “good parent” comparison (F = 12.10, df = 2; p<.001) after controlling for child age and family income. Fig 1 also shows the means across the three parental monitoring groups. Again, those adolescents who believed their parents needed to monitor more to meet the comparison reported the highest level of risk involvement in this sample (X = 3.45) compared to those who believed their parents needed to monitoring less (X = 2.95; p<.05) and those who believed their parents equally paired with that comparison (X = 1.96; p<.001).

Findings from the present study are among the first to highlight socio-cognitive factors that are associated with parents’ monitoring efforts and contribute to adolescents’ decisions to disclose information to their parents. For more than two decades, researchers have examined these types of factors with regard to parent-adolescent conflict. This work, more recently grounded in social comparison theoretical traditions [17], has documented differences between parent and adolescent expectations about adolescents’ behavioral autonomy [19], the timing of the resolution [20], and social conventions [21–22]. Collectively, this work has demonstrated that these factors directly contribute to differences in how parents and adolescents perceive a situation and how they, in turn, react to that situation. In this study, we illustrated that similar associations are found with regards to adolescents’ perceptions of their parents’ monitoring, their patterns of disclosure to their parents, the extent to which they perceive their parents’ monitoring knowledge, and their own risk behavior involvement.

Adolescents who perceived their parent as needing to provide more monitoring after comparing that parent to a “good parent” standard were more vulnerable to limited disclosure and subsequent risk than adolescents who perceived their parents to be similar to the “good parent” comparison. The patterns found in our findings are similar to those seen in the parenting style literature in that those adolescents who perceived their parents as needing to monitor more in this study may also be adolescents whose parents take on a more permissive parenting style. Likewise, adolescents who perceive their parents as needing to monitor less may represent those whose parents engage in authoritarian parenting. Both adolescent groups have consistently been shown to be more vulnerable to poor health outcomes [19, 23–24].

Identifying and understanding these and other contributing factors will be important for developing effective risk programming for adolescents and their families. Comparing a parents’ monitoring efforts to that of a “good parent” not only distinguished differences in adolescent disclosure but also adolescent perceptions of their parents’ monitoring knowledge and the extent to which adolescents were involved in a variety of risk behaviors.

## Conclusions

The findings from this study offer preliminary highlights about social comparisons adolescents may make that contribute to their willingness to disclose information to their parents and their decisions to engage in particular risk behaviors. However, this study is not without limitations. First, while not intended, the sample in this study is predominantly composed of females and of adolescents with very limited risk behavior involvement. In addition, the original intention of the baseline survey was not focused on cognitive processes. Thus, more work, with more advanced measurement of such processes, is needed in future studies. Given the cross-sectional design of this study, we are also not able to assess causality among the study variables.

Future work should further explore adolescents’ social comparisons or definitions of “good parent” monitoring before we can fully understand the nature and magnitude of the associations among these comparisons and adolescent behaviors. At this time, it is unclear whether adolescent perceptions of “good parent” monitoring are consistent, what factors contribute to that comparison, and how their perceptions of a “good parent” might differ as compared to parents’ perceptions. The results of this study illustrate that a minority of adolescents perceive their parents to fall short of a “good parent” standard in terms of monitoring behavior. This suggests that parents of adolescents may also benefit from structured parent training interventions that provide education regarding adolescents’ expectations of good parental monitoring behavior and teach parents how to effectively engage in this level of monitoring.

Researchers and clinicians may want to consider how to utilize evidence-based parent training models targeting child behavior problems to develop developmentally appropriate interventions aimed specifically at teaching effective parental monitoring strategies. Many parenting interventions designed to improve parenting skills are based on findings from the parenting styles literature. These interventions such as *Incredible Years* [25]; *Parent Management Training Oregon Model* (PMTO) [26]; and *Parent-Child Interaction Therapy* (PCIT) [27] target mainly parents of younger (elementary aged) children and are considered evidence-based treatments for child disruptive behavior problems [28]. Training components related to parental monitoring for both older children and their parents would fill an existing developmental gap toward the prevention of adolescent risk behaviors and unhealthy outcomes.

## Supporting Information

S1 Fig Questionnaire. Specific items used for the purpose of analyzing this study.
(PDF)
