# Supplementary material for: Do Parents Meet Adolescents’ Monitoring Standards? Examination of the Impact on Teen Risk Disclosure and Behaviors if They Don’t
Source: PLoS One. 2015 May 8;10(5):e0125750. doi: 10.1371/journal.pone.0125750 (PMC4425540; doi:10.1371/journal.pone.0125750)
Supplement: S1 Fig — Specific items used for the purpose of analyzing this study. (PDF) [file pone.0125750.s001.pdf]

Draft

Directions: Please indicate your response by filling in the appropriate circle.

Compared to what a "good" parent should do, do you think your parent should monitor or check-up on the following more, less, or about the same amount as he/she does now	More	Less	About the Same
1. My telephone usage	O	O	O
2. My appearance	O	O	O
3. My mood	O	O	O
4. My school work and performance	O	O	O
5. My activities and plans with friends	O	O	O
6. My health	O	O	O
7. My use of certain materials (e.g., magazines, CDs, books)	O	O	O
8. My television watching/usage	O	O	O
9. My use of money	O	O	O

Please answer the following questions by filling in the appropriate circle. If these situation DO NOT APPLY to you then please mark "Does not apply" for those particular questions.

Compared to what a "good" parent should do, do you think your parent should monitor or check-up on the following more, less, or about the same amount as he/she does now	Does not apply	More	Less	About the same
10. My computer usage	O	O	O	O
11. My driving	O	O	O	O
12. My activities and plans with boyfriends/girlfriends	O	O	O	O

Draft

Directions: Please indicate your response by filling in the circle.

How many times does your parent know who you are with on typical:	Never	A Few Times	Several Times	All the Time
1. School afternoons until 5pm	O	O	O	O
2. School evenings	O	O	O	O
3. Non-school days (weekends, holidays)	O	O	O	O
4. Non-school evenings (weekends, holidays)	O	O	O	O

How many times does your parent know where you are on typical:	Never	A Few Times	Several Times	All the Time
1. School afternoons until 5pm	O	O	O	O
2. School evenings	O	O	O	O
3. Non-school days (weekends, holidays)	O	O	O	O
4. Non-school evenings (weekends, holidays)	O	O	O	O

How many times does your parent know what you are doing on typical:	Never	A Few Times	Several Times	All the Time
1. School afternoons until 5pm	O	O	O	O
2. School evenings	O	O	O	O
3. Non-school days (weekends, holidays)	O	O	O	O
4. Non-school evenings (weekends, holidays)	O	O	O	O

Draft

Directions: Please indicate your response by filling in the appropriate circle.

In the PAST 4 months, have you done any of the following:	Yes	No
1. Drunk alcohol	O	O
2. Used tobacco (smokeless or cigarettes)	O	O
3. Smoked marijuana	O	O
4. Tried other drugs (other than marijuana)	O	O
5. Experienced a change in your mood that concerned your parent	O	O
6. Skipped school	O	O
7. Been suspended or in trouble at school	O	O
8. Had sexual intercourse without using condoms	O	O
9. Had sexual intercourse using condoms	O	O
10. Had sexual experiences, but you are still a virgin (you have not had intercourse or "gone all the way")	O	O
11. Been arrested or picked up by police	O	O
12. Lied about your activities	O	O
13. Stolen anything	O	O
14. Vandalized property	O	O
15. Stayed out past curfew	O	O
16. Snuck out of the house	O	O
17. Taken car without permission	O	O
18. Used family finances (e.g., credit card, cash, check) without permission	O	O

Draft

Directions: Please indicate your response by filling in the appropriate circle.

In the PAST 4 months, have you done any of the following:	Yes	No
19. Gone somewhere without parent's permission	O	O
20. Possessed materials that were against your parent's rules (e.g., music, movies)	O	O
21. Worked with friends to get around the rules	O	O
22. Received poor grades at school	O	O
23. Tried to look at things on the computer that would concern your parent (porn, etc)	O	O
24. Hung out with the "wrong" kids	O	O
25. Hung out with people your parent didn't know	O	O
26. Worn clothing or makeup that your parent thought was inappropriate	O	O
27. Gone without bathing for many days	O	O
28. Eaten unhealthy foods or not enough healthy foods	O	O
29. Used birth control pills	O	O
30. Used instant messaging or email to talk to someone who your parent does not approve	O	O
31. Used instant messaging or email to talk about things your parent does not approve	O	O
32. Gambled (played cards, games for money, slot machines, bet on events, etc.)	O	O

Draft

How much do you agree or disagree with each statement?	Strongly Agree	Agree	Disagree	Strongly Disagree
1. If my parent did not ask me questions, he/she would not know what I was doing.	O	O	O	O
2. I tell my parent what I am doing before he/she has to ask.	O	O	O	O
3. Only fathers or other male adults should discuss issues about sex with boys	O	O	O	O
4. Only mothers or other female adults should discuss issues about sex with girls	O	O	O	O
5. Parents should discuss issues about sexual intercourse with their kids before puberty	O	O	O	O
6. Parents should not initiate discussions about sex with their kids - only answer kids' questions about sex when asked	O	O	O	O
7. I will not have sexual intercourse until I get married	O	O	O	O
8. I feel it is important for my parent to know what my friends and I are doing while I am in my home	O	O	O	O
9. It is important for parents to ask for details when kids are spending time with their friends outside of the home	O	O	O	O
10. When a kid has been in trouble, it changes the way parents monitor that kid in the future	O	O	O	O
11. It is important for a parent to know where his/her child is all of the time	O	O	O	O
12. Kids work together to keep their activities secret	O	O	O	O
13. Teenagers will experiment with sex	O	O	O	O
14. As kids get older, they will experiment with alcohol and drugs	O	O	O	O
15. As kids get older, it is important for parents to respect their privacy by not asking questions about their plans	O	O	O	O

Draft

How much do you agree or disagree with each statement?	Strongly Agree	Agree	Disagree	Strongly Disagree
16. Trusting kids means not asking them about whom they are with, what they are planning to do, and where they go	O	O	O	O
17. Trusting kids means not checking on them while they are out	O	O	O	O
18. Trusting kids means not having them check in while they are out of the house	O	O	O	O
19. No matter how much information a parent has, a kid will still do things that are risky	O	O	O	O
20. People in my community help keep my parent informed about what I do	O	O	O	O
21. It is important for parents to have a good relationship with their kid's friends	O	O	O	O
22. Parents must seek out information about their kids because they won't hear about it from others (including their own children)	O	O	O	O
23. My parent doesn't trust me if he/she asks questions about my plans	O	O	O	O
24. Both of my parents receive the same information from me about my plans	O	O	O	O
25. The more my parent knows about my experiences, friends, and plans the less likely I am to have sex, use drugs, etc	O	O	O	O
26. Parents should always meet their kid's girlfriends/boyfriends	O	O	O	O
27. Kids should always have a curfew while they live with their parents	O	O	O	O
28. Parents should not change their set rules for any reason	O	O	O	O

Draft

How much do you agree or disagree with each statement?	Strongly Agree	Agree	Disagree	Strongly Disagree
29. Kids should talk to their parents if something is wrong	O	O	O	O
30. Kids should manage their money on their own	O	O	O	O
31. It is alright for parents to buy alcohol for their kids	O	O	O	O
32. It is alright for parents to provide cigarettes for their kids	O	O	O	O
33. It is alright for parents to provide marijuana or other drugs for their kids	O	O	O	O
34. If kids want to do something parents can't stop them	O	O	O	O
35. My neighbors and/or other community members will keep an eye on what I do	O	O	O	O
36.Someone in my community would tell my parent if I were doing something he/she didn't appprove of	O	O	O	O
37. Stepchildren are monitored differently than biological children	O	O	O	O
38. Stepchildren have to follow the rules more than biological children	O	O	O	O
39. It's harder for parents to monitor stepchildren than biological children	O	O	O	O
40. Parents would be comfortable talking to stepchildren about sensitive topics	O	O	O	O
41. Parents of a (step) blended family argue over how to effectively monitor their children	O	O	O	O
42. Girls should be monitored more than boys	O	O	O	O
43. Younger kids should be monitored more than older kids	O	O	O	O

Draft

How much do you agree or disagree with each statement?	Somewhat Agree	Agree	Disagree	Strongly Disagree
44. I can discuss my beliefs with my parent without feeling embarrassed or restrained	O	O	O	O
45. Sometimes I have trouble believing everything my parent tells me	O	O	O	O
46. My parent is always a good listener	O	O	O	O
47. I am sometimes afraid to ask my parent for what I want	O	O	O	O
48. My parent has a tendency to say things to me, which would be better left unsaid	O	O	O	O
49. My parent can tell how I'm feeling without asking	O	O	O	O
50. I am very satisfied with how my parent and I talk together	O	O	O	O
51. If I were in trouble I could tell my parent	O	O	O	O
52. I openly show affection to my parent	O	O	O	O
53. When we are having a problem, I often give my parent the silent treatment	O	O	O	O
54. I am careful what I say to my parent	O	O	O	O
55. When I talk to my parent, I say things that would be better left unsaid	O	O	O	O
56. When I ask questions, my parent gives honest answers	O	O	O	O
57. My parent tries to understand my point of view	O	O	O	O
58. There are topics I avoid discussing with my parent	O	O	O	O
59. I find it easy to discuss problems with my parent	O	O	O	O
60. It is very easy for me to express all my true feelings to my parent	O	O	O	O
61. My parent nags/bothers me	O	O	O	O
62. My parent insults me when he/she is angry with me	O	O	O	O
63. I don't think I can tell my parent how I feel about some things	O	O	O	O

Draft

How much do you agree or disagree with each statement?	Strongly Agree	Agree	Disagree	Strongly Disagree
64. My parent expects me to call if I am going to be late	O	O	O	O
65. I tell my parent who I am going to be with	O	O	O	O
66. I talk to my parent about plans with friends	O	O	O	O
67. When I go out my parent asks me where I am going	O	O	O	O
68. My parents work as a team to monitor me	O	O	O	O
69. My parents have different approaches to monitoring me	O	O	O	O
70. Parents should expect their kids to experiment with risky situations	O	O	O	O
71. I will follow my parent's rules because I know he/she is usually right about things	O	O	O	O
72. It is ok for parents to go through their kids' drawers and closets	O	O	O	O
73. If my parent knew everything I did he/she would be upset	O	O	O	O
74. It is easy to get something by my parent and not get caught	O	O	O	O
75. Parents don't know everything about their kid's activities and friends even if they think they do	O	O	O	O
76. My parent doesn't need to know what I look at on the internet	O	O	O	O
77. My parent doesn't need to know who I talk to on the computer	O	O	O	O
78. Many kids don't care what their parent has to say	O	O	O	O
79. Many parents do things that they tell their children not to do	O	O	O	O
80. If someone can find the answers to what is troubling me, my parent is the one	O	O	O	O
81. My parent honestly believes that they have the skills necessary to be a good parent	O	O	O	O
